# AKR1C3-Mediated Adipose Androgen Generation Drives Lipotoxicity in Women With Polycystic Ovary Syndrome

**DOI:** 10.1210/jc.2017-00947

**Published:** 2017-06-22

**Authors:** Michael W. O’Reilly, Punith Kempegowda, Mark Walsh, Angela E. Taylor, Konstantinos N. Manolopoulos, J. William Allwood, Robert K. Semple, Daniel Hebenstreit, Warwick B. Dunn, Jeremy W. Tomlinson, Wiebke Arlt

**Affiliations:** 1 Institute of Metabolism and Systems Research, University of Birmingham, Edgbaston, Birmingham B15 2TT, United Kingdom; ^2^Centre for Endocrinology, Diabetes and Metabolism, Birmingham Health Partners, Edgbaston, Birmingham B15 2TH, United Kingdom; 3 School of Life Sciences, University of Warwick, Coventry CV4 7AL, United Kingdom; 4 School of Biosciences, University of Birmingham, Edgbaston, Birmingham B15 2TT, United Kingdom; 5 The University of Cambridge Metabolic Research Laboratories, Wellcome Trust-Medical Research Council Institute of Metabolic Science, Cambridge CB2 1TN, United Kingdom; 6 Phenome Centre Birmingham, University of Birmingham, Edgbaston, Birmingham B15 2TT, United Kingdom; 7 Oxford Centre for Diabetes, Endocrinology and Metabolism, National Institutes of Health Research (NIHR) Biomedical Research Centre, University of Oxford, Churchill Hospital, Oxford OX3 7LE, United Kingdom; 8 NIHR Birmingham Liver Biomedical Research Unit, University of Birmingham, Birmingham B15 2TT, United Kingdom

## Abstract

**Context:**

Polycystic ovary syndrome (PCOS) is a prevalent metabolic disorder occurring in up to 10% of women of reproductive age. PCOS is associated with insulin resistance and cardiovascular risk. Androgen excess is a defining feature of PCOS and has been suggested as causally associated with insulin resistance; however, mechanistic evidence linking both is lacking. We hypothesized that adipose tissue is an important site linking androgen activation and metabolic dysfunction in PCOS.

**Methods:**

We performed a human deep metabolic *in vivo* phenotyping study examining the systemic and intra-adipose effects of acute and chronic androgen exposure in 10 PCOS women, in comparison with 10 body mass index–matched healthy controls, complemented by *in vitro* experiments.

**Results:**

PCOS women had increased intra-adipose concentrations of testosterone (*P* = 0.0006) and dihydrotestosterone (*P* = 0.01), with increased expression of the androgen-activating enzyme aldo-ketoreductase type 1 C3 (AKR1C3) (*P* = 0.04) in subcutaneous adipose tissue. Adipose glycerol levels in subcutaneous adipose tissue microdialysate supported *in vivo* suppression of lipolysis after acute androgen exposure in PCOS (*P* = 0.04). Mirroring this, nontargeted serum metabolomics revealed prolipogenic effects of androgens in PCOS women only. *In vitro* studies showed that insulin increased adipose AKR1C3 expression and activity, whereas androgen exposure increased adipocyte *de novo* lipid synthesis. Pharmacologic AKR1C3 inhibition *in vitro* decreased *de novo* lipogenesis.

**Conclusions:**

These findings define an intra-adipose mechanism of androgen activation that contributes to adipose remodeling and a systemic lipotoxic metabolome, with intra-adipose androgens driving lipid accumulation and insulin resistance in PCOS. AKR1C3 represents a promising therapeutic target in PCOS.

## Teaser

This integrated *in vivo* and *in vitro* study examines the role of AKR1C3 in adipose androgen generation in PCOS, and how locally activated androgens exert lipotoxic effects on adipose lipid metabolism.

Polycystic ovary syndrome (PCOS) is a prevalent disorder in women of reproductive age, and is defined by androgen excess (AE) and anovulatory infertility (1, 2). It affects 6.1% to 19.9% of women, depending on diagnostic criteria applied (3). In terms of clinical consequences, however, PCOS is primarily a disorder with adverse metabolic risk impact, as evidenced by a high prevalence of insulin resistance (4), dyslipidemia (5), and increased rates of type 2 diabetes mellitus and hypertension (3, 6), over and above those observed in simple obesity. Recent data also highlight increased rates of cardiovascular disease (7) and nonalcoholic fatty liver disease (8, 9) in women affected by PCOS.

AE is a defining feature of PCOS (10); however, its origins remain insufficiently understood. Although traditionally perceived as a disorder of purely ovarian origin, it is now clear that there are also complex contributions from the adrenal and peripheral tissues to PCOS-related AE (11–14). The potent androgens testosterone (T) and 5*α*-dihydrotestosterone (DHT) can be activated from androgen precursors in peripheral target tissues of androgen action (12, 15). The severity of AE and insulin resistance in PCOS is closely correlated, but the direction of causality remains unclear. Insulin resistance because of monogenic insulin receptor mutations has been shown to be associated with a severe AE phenotype (16). Conversely, monogenic causes of AE are associated with adverse metabolic consequences and can present with a PCOS phenotype (17–19).

One of the major metabolic compartments, adipose tissue, is capable of androgen generation, a process that is tightly regulated by a complex network of activating and inactivating enzymes (20). The enzyme aldo-ketoreductase type 1C3 (AKR1C3), which converts androstenedione (A4) to the biologically active androgen T, is abundantly expressed in adipose tissue (21), with increased expression in subcutaneous fat from both women with simple obesity (22) and PCOS (23). Previous work has shown that AKR1C3 is the only enzyme expressed in adipose tissue that is capable of activating A4 to T (22). The enzyme 5*α*-reductase type 1 (SRD5A1) activates T in peripheral target tissues to the most potent androgen, DHT, and is highly expressed in adipose tissue (24). Studies in rodent and nonhuman primate models show that pre- and postnatal androgen exposure in women leads to increased adipocyte size, enlarged visceral fat depots, and reduced insulin sensitivity (25–27). However, human data on the effects of AE on key processes of adipose biology, such as adipogenesis and lipid metabolism, are lacking.

In this study, we explored the hypothesis that increased intra-adipose androgen generation by AKR1C3 is a central driver of adipose tissue dysfunction and lipotoxicity in PCOS. To test our hypothesis, we undertook a study using innovative *in vivo* physiology tools for deep metabolic phenotyping in a cohort of women with PCOS and age-, sex-, and body mass index (BMI)–matched healthy controls, alongside *in vitro* experiments defining the impact of androgens on human adipocyte biology.

## Methods

### Study participants

**Figure 1.**
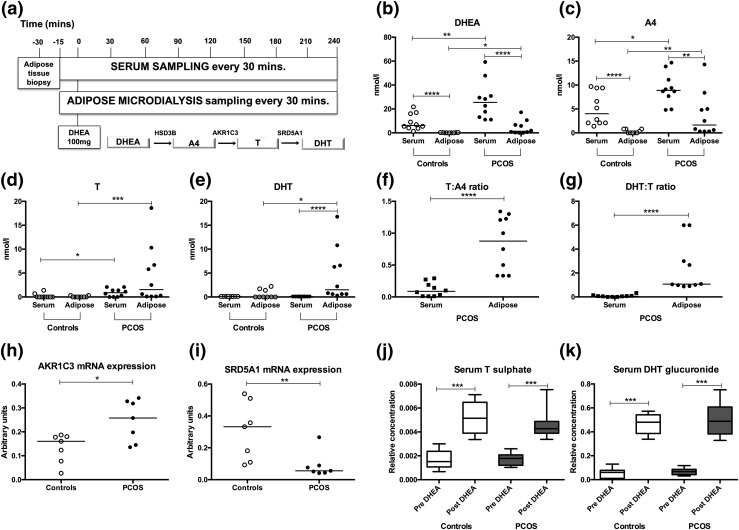
Circulating androgens and *in vivo* intra-adipose tissue androgen synthesis in PCOS (n = 10) and age- and BMI-matched healthy controls (n = 10). (a) Deep metabolic *in vivo* phenotyping protocol with serum and adipose microdialysate sampling every 30 minutes for 4 hours. After baseline sampling, an acute androgen challenge was administered by oral intake of a 100-mg dose of the androgen precursor DHEA at 0 minutes; schematic represents the classic androgen synthesis pathway from DHEA to active androgens. (b–e) Serum and *in vivo* intra-adipose concentrations of DHEA, A4, T, and 5*α*-DHT in controls (○) and PCOS women (●). Lines represent the median of each group. (f and g) Steroid ratios reflective of the conversion of A4 to T (T:A4) and the conversion of T to DHT (DHT:T) as measured in serum and adipose tissue microdialysate of PCOS women. Black squares represent PCOS serum. Lines represent the median of each group. (h and i) Relative messenger RNA expression of the androgen-generating enzymes AKR1C3 (converting A4 to T) and SRD5A1 (converting T to DHT) in subcutaneous abdominal adipose tissue from PCOS and controls (n = 7 for each). Lines represent the median of each group. (j and k) Serum T sulfate and DHT glucuronide concentrations before and 150 minutes after oral administration of the androgen precursor DHEA. Boxes represent median and 25th to 75th percentile, and whiskers represent 10th and 90th percentile. Significance levels: *P < 0.05; **P < 0.01; ***P < 0.001; ****P < 0.0001 (Mann-Whitney *U* test). All steroid concentrations were measured by mass spectrometry–based assays. mRNA, messenger RNA.

Women with PCOS aged between 18 and 40 years were recruited from outpatient clinics at University Hospitals Birmingham and Birmingham Women’s Hospital. Full ethical approval was obtained from Edgbaston Research Ethics Committee (reference no. 12/WM/0206) and all participants provided written informed consent prior to inclusion in the study. PCOS was diagnosed according to the Rotterdam European Society of Human Reproduction and Embryology 2004 criteria, with the presence of two or more of the following: oligo/anovulation (Anov), clinical signs of AE, and polycystic ovaries (PCOs) on ultrasound (10); however, only PCOS women with clinical or biochemical signs of AE were recruited to the *in vivo* study (phenotypes AE + Anov + PCO, AE + Anov, and AE + PCO). Other potential causes of oligomenorrhea and AE were excluded by history, physical examination, and biochemical assessment. All healthy controls were age- and BMI-matched, and recruited via local advertisement, with exclusion of PCOS on clinical and biochemical grounds. Recruitment numbers for each study group were agreed based on *a priori* power calculations; based on estimated detectable differences and standard deviations in adipose microdialysis data and serum androgen profiles across the dehydroepiandrosterone (DHEA) challenge, a sample size of 10 PCOS and 10 control patients was deemed sufficient to achieve 80% statistical power for the detection of a difference with *P* < 0.05. Exclusion criteria for the study were as follows: recent glucocorticoid treatment (within 3 months), pregnancy, age <18 or >45 years, recent oral contraceptive use (within 3 months), hyperprolactinemia, thyroid disorders, and dysglycemia (impaired fasting glucose, impaired glucose tolerance, or overt diabetes mellitus). PCOS women were also excluded if they had received prior treatment with metformin, insulin sensitizers, or androgen receptor blockers. Detailed information on the clinical protocol is provided in the Supplemental Methods and Fig. 1(a). Subcutaneous abdominal fat samples were obtained by excisional biopsy (2 to 3 cm^3^, ~3 to 5 g) in both PCOS and control women under local anesthetic and sterile conditions, 5 cm lateral to the umbilicus. Excised adipose tissue was immediately placed in RNALater (Life Technologies, Paisley, United Kingdom) and stored at −20°C for subsequent RNA extraction.

### Biochemical analysis

Insulin (Mercodia, Uppsala, Sweden) and serum-free fatty acids (Zen-Bio, Research Triangle Park, NC) were measured using commercially available assays, according to manufacturer instructions. Plasma glucose was measured using the 2300 STAT PLUS analyzer (YSI Inc., Life Sciences, OH). Homeostasis model assessment of insulin resistance (HOMA-IR) was calculated using the formula [fasting glucose (mmol/L) × fasting insulin (pmol/L)/135]. Microdialysate samples were collected in microvials and analyzed using a mobile photometric, enzyme-kinetic analyzer (CMA Iscus Flex; M Dialysis AB, Johannesov, Sweden) for glucose, pyruvate, lactate, and glycerol.

### Steroid metabolite analysis (adipose microdialysate, serum, and cellular supernatant)

Androgens from adipose microdialysate, serum, and cellular supernatant were measured by liquid chromatography/tandem mass spectrometry (LC-MS/MS) using an Xevo mass spectrometer with Acquity uPLC system (Waters, Herts, United Kingdom), as described previously (11). Measurement of T, A4, and DHEA was facilitated by serum steroid oxime analysis, and carried out in positive mode. T, A4, and DHEA were extracted from 150 μL interstitial fluid, 400 μL serum, and 1 mL cellular supernatant via liquid-liquid extraction with 1, 2, and 4 mL *tert*-butyl-methyl-ether, respectively. This was followed by derivatization into oximes. Serum dehydroepiandrosterone sulfate was extracted from 20 μL serum with 100 μL acetonitrile, before evaporation under constant nitrogen flow (28).

For each LC-MS/MS assay, chromatography was optimized using a methanol/water (0.1% formic acid) gradient system. Steroids were quantified according to a linear calibration series with appropriate internal standards. Individual steroids were identified by matching retention times and two mass transitions in comparison with a deuterated reference compound.

Measurement of urinary steroid metabolite excretion by gas chromatography mass spectrometry is described in the Supplemental Methods.

### Nontargeted serum metabolomics

We carried out mass spectrometry–based, nontargeted metabolome analysis in negative and positive ion modes at the Phenome Centre Birmingham, United Kingdom. In-depth methodology for nontargeted serum metabolomic profiling has been published by our group previously (24), and the full protocol is available in the Supplemental Methods.

### Primary human adipocyte culture

Paired primary subcutaneous preadipocytes were isolated from adipose tissue obtained from healthy, nondiabetic women aged 18 to 45 years undergoing elective abdominal laparotomies for nonmalignant, noninflammatory disease, using methods described previously (29). Samples from women with diabetes or treated with systemic glucocorticoids in the last 3 months were excluded. Sample collection was facilitated via the University of Birmingham Tissue Biorepository. Briefly, adipose tissue samples were dissected into 2- to 3-mm^3^ pieces and digested with type II collagenase (Sigma Aldrich, Dorset, United Kingdom) at 37°C for 60 minutes. Samples were then centrifuged at 12,000 rpm for 5 minutes. Preadipocytes were extracted from a pellet containing the stromovascular components and resuspended in Dulbecco’s modified Eagle medium/nutrient mixture F12, containing fetal bovine serum 10% (Sigma-Aldrich) and penicillin-streptomycin 1% (ThermoFisher Scientific, Warrington, United Kingdom). Preadipocytes were proliferated to confluence before chemically defined culture media was applied for 14 days to stimulate differentiation into adipocytes. Differentiation was initiated by washing the cells in serum-free media, followed by culture with differentiation media (Dulbecco’s modified Eagle medium–F12 media containing biotin 33 μM, pantothenate 17 μM, tri-iodothyronine 1 nM, insulin 167 nM, cortisol 1 μM, and rosiglitazone 1 μM). AKR1C3 activity was determined by generation of T after incubation of cells with 200 nM of the T precursor A4 in serum-free media for 24 hours.

### Simpson-Golabi-Behmel syndrome preadipocyte cell line culture

Conditions for culture and differentiation of the human preadipocyte Simpson-Golabi-Behmel syndrome (SGBS) cell line are described in the Supplemental Appendix.

### Functional studies of lipid metabolism

A detailed description of methodology for *de novo* lipogenesis, *β* oxidation, and free fatty acid uptake is provided in the Supplemental Appendix.

### Statistics

SPSS version 22 (SPSS, Chicago, IL) was used for data analysis. Data are presented as mean ± standard error of the mean unless otherwise stated. Baseline clinical and biochemical data were not normally distributed and are therefore presented as median and interquartile range. Area under the curve (AUC) analysis was carried out using the trapezoidal method. For comparison of single variables, *t* tests (paired or unpaired as appropriate) were used, or nonparametric equivalents where data were not normally distributed. One-way analysis of variance with *post hoc* Tukey testing was used for multiple comparisons between different groups. For real-time polymerase chain reaction data, statistical analysis was performed on mean *Δ*ct values only. Correlation testing was performed using Pearson correlation coefficient or Spearman test as appropriate. Serum metabolomics data were analyzed by applying univariate (Mann-Whitney *U* test or Wilcoxon signed-rank test) analysis after normalization to total peak area per sample.

## Results

### Systemic androgen concentrations and measures of insulin resistance

Ten women with PCOS and 10 healthy, sex-, age-, and BMI-matched volunteers were recruited to an *in vivo* physiology study for deep metabolic phenotyping [see Fig. 1(a) and for detailed protocol description the Supplemental Appendix]; clinical and biochemical characteristics of both groups are summarized in Supplemental Table 1.

Baseline circulating androgens, measured by LC-MS/MS, were all significantly higher in PCOS than in controls, as was insulin resistance assessed by HOMA-IR (*P* = 0.003) (Supplemental Table 1). Similarly, truncal fat mass and volume on body composition imaging by dual x-ray absorptiometry were increased in the PCOS group compared with matched controls (Supplemental Table 1). These findings demonstrate the expected systemic AE, insulin resistance, and increased visceral adiposity in PCOS subjects in comparison with sex-, age-, and BMI-matched controls.

### Adipose tissue-specific AE in PCOS

To explore *in vivo* androgen concentrations in the adipose tissue metabolic compartment, we inserted a microdialysis catheter into the abdominal subcutaneous adipose tissue of each participant, which allowed the sampling of adipose tissue interstitial fluid across a semipermeable membrane. We used LC-MS/MS to measure androgens in adipose microdialysate in both PCOS and control subjects, representative of *in vivo* intratissue androgen homeostasis.

Adipose tissue concentrations of the androgen precursors DHEA (*P* = 0.01) and A4 (*P* = 0.01) and the active androgen T (*P* = 0.0006) were significantly increased in PCOS subjects [Fig. 1(b–d)]. The most potent androgen DHT was quantifiable in adipose interstitial fluid in all 10 PCOS patients and in six of the 10 control patients, with significantly higher concentrations in PCOS (4.5 ± 1.7 *vs* 0.9 ± 0.3 nmol/L, *P* = 0.01) [Fig. 1(e)]. Adipose tissue microdialysate concentrations of DHEA, A4, and DHT all correlated positively with HOMA-IR (*R* = 0.51, *P* = 0.02, *R* = 0.54, *P* = 0.01 and *R* = 0.44, *P* = 0.04, respectively), whereas intra-adipose T correlated positively with fasting insulin levels (*R* = 0.49, *P* = 0.04) (for complete correlation analysis, see Supplemental Table 2).

Of note, concentrations of the androgen precursors DHEA and A4 were higher in serum (*i.e.*, in systemic circulation) than in adipose tissue in both PCOS and control women [Fig. 1(b) and 1(c)]. By contrast, intra-adipose concentrations of the androgen receptor-activating androgens T and DHT were significantly higher than serum levels in PCOS women (*P* < 0.0001) [Fig. 1(d) and 1(e)]. Androgen concentrations in control women did not differ between circulation and adipose tissue.

The intra-adipose ratio of T to A4, reflective of AKR1C3 activity, was significantly higher than in serum (*P* < 0.0001) [Fig. 1(f)] in the PCOS patients but not in the BMI-matched controls (data not shown). Similarly, in the PCOS women, the intra-adipose ratio of DHT to T, reflecting 5*α*-reductase activity, was significantly higher than in serum (*P* < 0.0001) [Fig. 1(g)].

Subcutaneous adipose tissue biopsies from PCOS and control women were used for messenger RNA expression analysis by real-time quantitative polymerase chain reaction. This showed significantly increased AKR1C3 messenger RNA expression in subcutaneous fat from PCOS women (mean *Δ*ct, 12.1 ± 0.2 in PCOS *vs* 13.1 ± 0.4 in controls, *P* = 0.04) [Fig. 1(h)]. Conversely, we found significantly decreased intra-adipose expression of SRD5A1 in PCOS women (mean *Δ*ct, 16.1 ± 0.4 in PCOS *vs* 14.3 ± 0.4 in controls, *P* = 0.005) [Fig. 1(i)], indicating that AKR1C3 is the major driver of intra-adipose AE in PCOS.

### Acute oral androgen challenge suppresses *in vivo* lipolysis in PCOS adipose tissue

To examine the effect of androgens on *in vivo* adipose tissue metabolism, study participants received an oral dose of the androgen precursor DHEA at baseline, followed by sampling of serum and adipose tissue microdialysate every 30 minutes for 4 hours. We have previously shown that this DHEA challenge test results in rapid generation of downstream androgens that can be detected in serum and urine (15, 30). After the oral ingestion of 100 mg DHEA, we measured circulating androgens by LC-MS/MS and documented significantly higher AUCs (mean ± standard error of the mean, nmol/L·min) in women with PCOS for serum DHEA (7437 ± 720 *vs* 4317 ± 654 in controls, *P* = 0.005), A4 (258 ± 65 *vs* 63 ± 25 in controls, *P* = 0.01), and T (2507 ± 237 *vs* 1556 ± 303 in controls, *P* = 0.01). Increased circulating concentrations of T sulfate and DHT glucuronide after DHEA exposure in both controls and PCOS (all *P* < 0.0001) [Fig. 1(j) and 1(k)] further documented the extent of acute androgen generation after DHEA administration. This demonstrates that we exposed both groups to an equivalent acute androgen challenge on the background of chronic AE (PCOS) or normal circulating androgen concentrations (controls).

**Figure 2.**
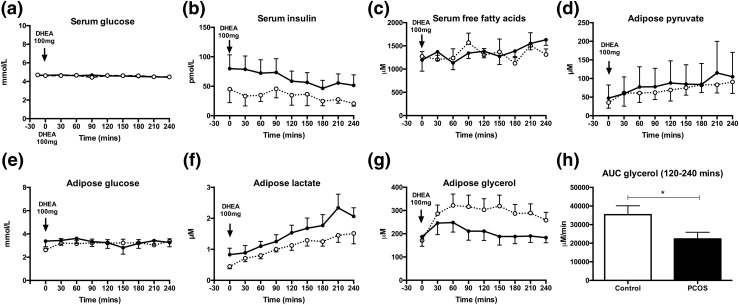
Impact of acute oral androgen challenge (DHEA 100 mg) on *in vivo* metabolic markers in serum and adipose tissue microdialysate. Serum glucose, insulin, and free fatty acids (a–c) and intra-adipose pyruvate, glucose, lactate, and glycerol levels (d–g) in PCOS (n = 10, dark line, ●) and control women (n = 10, dotted line, ○ across the DHEA challenge test) (mean ± standard error of the mean). Time of oral administration of 100 mg DHEA indicated by arrow. (h) AUC for glycerol between 120 and 240 minutes after androgen exposure in the PCOS group compared with controls. *P < 0.05 (Mann-Whitney *U* test). For further details see Supplemental Methods.

To examine the *in vivo* metabolic impact of this androgen load, we measured markers of insulin sensitivity and lipid metabolism at baseline and after acute DHEA exposure, looking at both systemic (serum) and tissue-specific (adipose microdialysate) outcomes (Fig. 2). Plasma glucose levels were similar at baseline and across the DHEA challenge in both PCOS and controls [Fig. 2(a)]. Baseline fasting insulin was significantly increased in PCOS (Supplemental Table 1), but AUC values for insulin across the DHEA challenge (pmol/L·min) did not differ significantly between PCOS and controls (14,672 ± 4120 *vs* 8043 ± 2958, *P* = 0.21) [Fig. 2(b)]. There was no difference in baseline or AUC values for serum-free fatty acids between the two groups [Fig. 2(c)].

Assessing the adipose tissue-specific *in vivo* metabolic response, we measured metabolic markers in adipose microdialysate. Adipose pyruvate, lactate, and glucose did not differ significantly between PCOS women and controls at baseline and in response to DHEA [Fig. 2(d–f)]. However, AUC values for glycerol (μM·min) significantly decreased in PCOS patients after DHEA (35,347 ± 4781 in PCOS *vs* 22,310 ± 3577 in controls, *P* = 0.04) [Fig. 2(g) and 2(h)], consistent with decreased lipid mobilization in PCOS in response to acute androgen exposure.

### AE induces distinct changes in the circulating metabolome in PCOS

**Figure 3.**
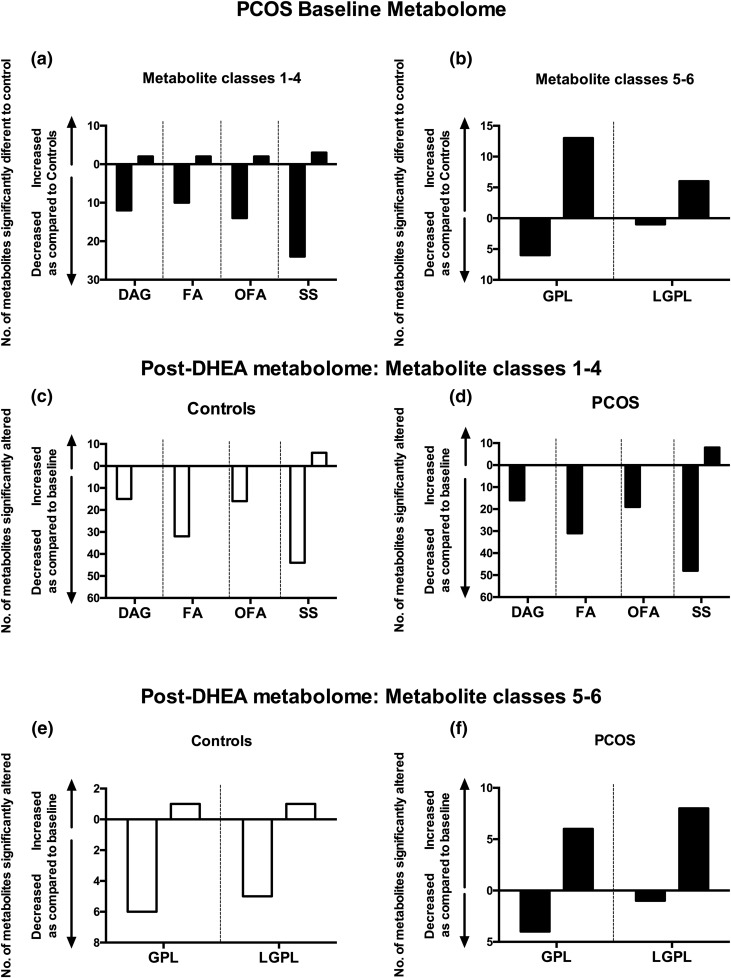
Baseline differences and changes induced in the nontargeted serum metabolome by acute androgen exposure in women with PCOS (n = 10) and age- and BMI-matched healthy control women (n = 10). (a) The number of serum metabolites associated with lipid and steroid metabolism observed to be significantly different (*P* < 0.01) at baseline between control and PCOS subjects. (b) Significantly different GPL and LGPL metabolites between PCOS women and controls at baseline. (c and d) Metabolic responses lipid and steroid metabolite after exposure of controls and PCOS subjects to an acute androgen challenge (DHEA 100 mg administered orally at 0 minutes; serum metabolome analysis carried out with 150-minute serum sample, representative of the maximum of circulating androgen concentrations after DHEA). (e and f) Differential response of GPLs and LGPLs to the acute DHEA challenge in comparison of controls and PCOS women. Data matrix analyzed applying univariate (Wilcoxon signed-rank test) after normalization to total ion current per sample. DAG, diacylglyceride; FA, fatty acid; OFA, oxidized fatty acids; SS, sterols and steroid metabolites.

Mass spectrometry–based nontargeted metabolome analysis was used to determine differences in global metabolic phenotype in PCOS serum compared with controls before and after DHEA administration. At baseline, we identified 119 metabolites that were statistically different (*P* < 0.01) when comparing PCOS with control subjects. Significantly perturbed metabolite classes in PCOS included diacylglycerides, fatty acids and oxidized fatty acids, sterol and steroid metabolites, and glycerophospholipids (GPLs) and lysoglycerophospholipids (LGPLs) [Fig. 3(a) and 3(b)]; other classes included aromatic amino acid metabolism, ceramides and sphingolipids, and ubiquinone/quinone metabolism. Both oxidized and nonoxidized fatty acids and most diacylglycerides showed higher concentrations in control subjects, whereas GPL and LGPL concentrations were higher in PCOS subjects.

In response to DHEA administration (*i.e.*, acute androgen exposure), the metabolome of PCOS patients (with a background of chronic AE) and that of BMI-matched controls (with normal baseline androgens) showed distinct changes [Fig. 3(c–f); Supplemental Table 3]. DHEA induced highly substantial changes in 15 diacylglycerides, 32 fatty acids, and 16 oxidized fatty acids in the control group; in PCOS women, changes were observed in 16 diacylglycerides, 31 fatty acids, and 19 oxidized fatty acids. In all cases, DHEA reduced circulating concentrations of diacylglycerides and oxidized and nonoxidized fatty acids compared with baseline, in both PCOS and controls, a demonstration of the potent effects of androgens in the regulation of lipid metabolism.

Of note, most GPLs and LGPLs, which were significantly increased in PCOS at baseline [Fig. 3(b)], showed a diametrically opposed metabolic response to the acute androgen challenge, with substantial further upregulation in PCOS and downregulation in the BMI-matched healthy controls [Fig. 3(e) and 3(f); Supplemental Table 3], indicating a distinctly different metabolic response to androgens in PCOS.

### Androgens promote adipose lipid accumulation *in vitro*

**Figure 4.**
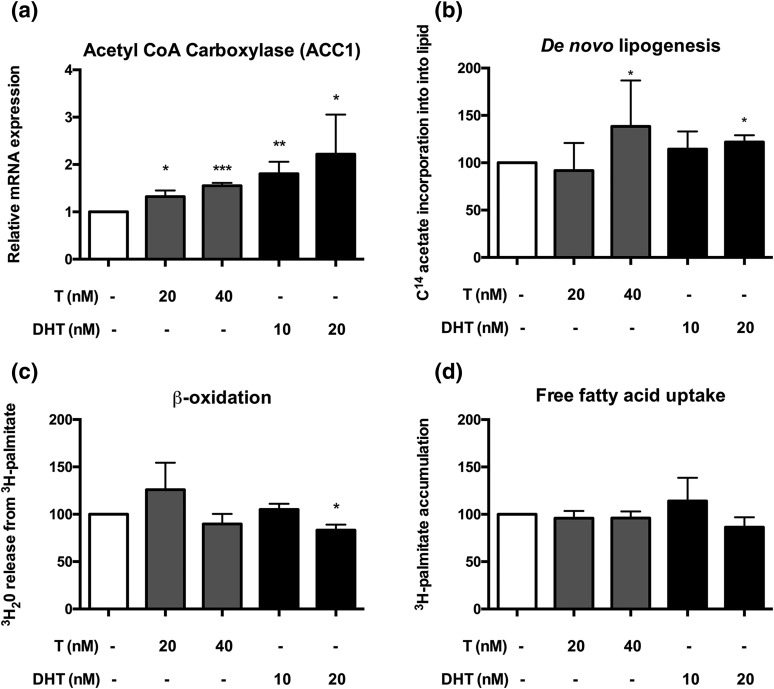
The effects of androgens on adipose lipid metabolism in the human preadipocyte SGBS cell line. (a) The effect of androgens (T, light-shaded bars; 5*α*-DHT, dark-shaded bars) on the messenger RNA expression of ACC1, the main provider of malonyl-CoA for fatty acid synthesis. (b) Effect of T and DHT on *de novo* lipogenesis, determined by incorporation of ^14^C acetate into lipid. (c) Effects of T and DHT on *β* oxidation, determined by ^3^H_2_0 release from ^3^H-palmitate. (d) Effects of androgens on free fatty acid uptake. All data are presented as the mean ± standard error of the mean of the three to five experiments. Significance levels: *P < 0.05; **P < 0.01; ***P < 0.001 compared with control (analysis of variance with *post hoc* Tukey test). mRNA, messenger RNA.

To dissect the observed effects in further detail, we used a human preadipocyte cell line, SGBS, previously validated as a model of human subcutaneous adipocyte biology (31). To determine the role of androgens in adipose lipid metabolism, differentiated adipocytes were treated with increasing concentration of T and DHT. Androgen treatment of 24 hours resulted in a dose-dependent increase in messenger RNA expression of acetyl-CoA-carboxylase (ACC) 1, the rate-limiting step in lipogenesis, but not ACC2 (fold change *vs* untreated control: T 20 nM, 1.3 ± 0.1; *P* = 0.02; T 40 nM, 1.6 ± 0.1; *P* < 0.001; DHT 10 nM, 1.8 ± 0.3; *P* = 0.006; DHT 20 nM, 2.2 ± 0.8; *P* = 0.01) [Fig. 4(a)].

Consistent with upregulation of ACC1 messenger RNA expression, T 40 nM significantly increased *de novo* lipogenesis in SGBS cells in the absence of insulin, as measured by uptake of 1-[^14^C]-acetate incorporation into lipid (150.4% ± 7.3% of untreated cells, *P* = 0.02) [Fig. 4(b)]. This effect was also observed with DHT 20 nM (121.9% ± 8.4% of untreated cells, *P* = 0.03), confirming it as androgen-mediated as DHT, in contrast with T, cannot be converted to estrogens via aromatase activity. Insulin (5 nM) expectedly resulted in a significant increase of *de novo* lipogenesis compared with control incubations without insulin (250.0% ± 19.1%, *P* = 0.009). However, there was no additional effect of insulin when added to the incubations with androgens (*P* = 0.9 for both T and DHT).

In SGBS cells, exposure to DHT 20 nM significantly decreased *β* oxidation of fatty acids compared with untreated control cells (83.2% ± 5.9%, *P* = 0.03) [Fig. 4(c)], whereas we did not observe androgen effects on free fatty acid uptake [Fig. 4(d)].

### AKR1C3 links adipose AE and lipid accumulation in PCOS

To examine the role of insulin in AKR1C3-mediated androgen generation, we examined expression and activity of AKR1C3 in primary human adipocytes and SGBS cells before and after insulin treatment. AKR1C3 messenger RNA expression increased significantly across differentiation from preadipocyte (day 0) to the mature adipocyte (day 14) phenotype in both subcutaneous and omental female adipocytes (mean *Δ*ct for subcutaneous fat, 16.4 ± 0.5 for preadipocytes *vs* 11.7 ± 1.3 for adipocytes; *P* = 0.03; mean *Δ*ct for omental fat, 19.5 ± 0.1 for preadipocytes *vs* 11.4 ± 1.1 for adipocytes; *P* = 0.001). AKR1C3 activity, as determined by generation of T after incubation of cells with 200 nM A4 for 24 hours, was significantly upregulated across adipocyte differentiation in both subcutaneous and omental fat depots (*P* = 0.02 and *P* = 0.04, respectively).

**Figure 5.**
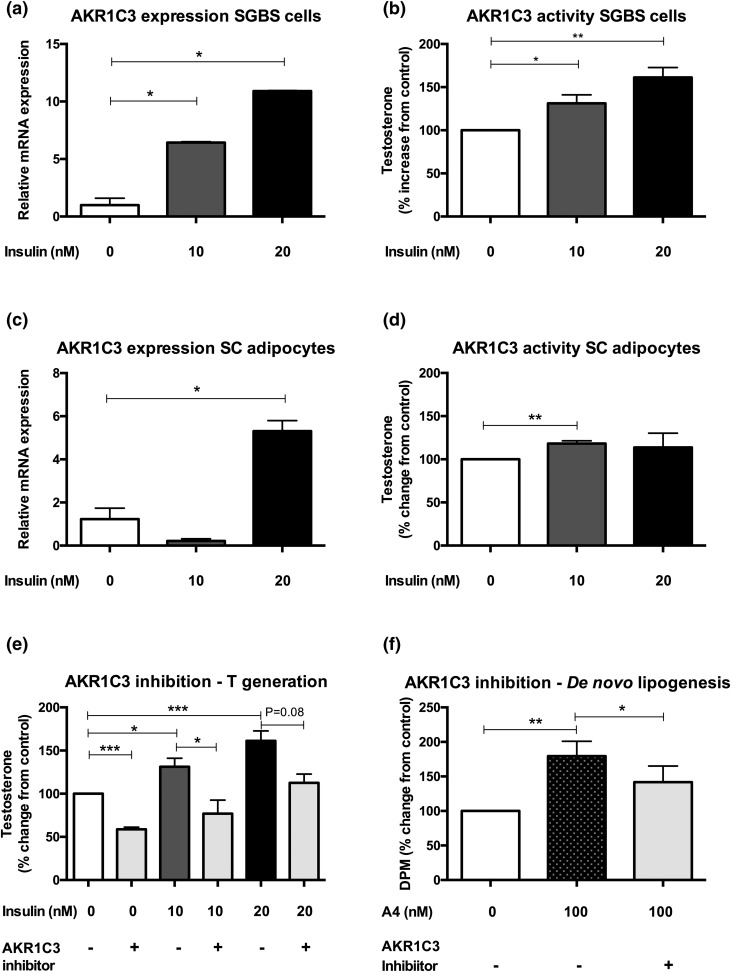
Expression, activity, and inhibition of the androgen-activating enzyme AKR1C3 in subcutaneous adipose tissue. (a–d) Expression and activity of AKR1C3 with and without insulin stimulation in the human preadipocyte SGBS cell line and primary subcutaneous adipocytes from women undergoing elective surgery. (e) Effect of pharmacologic AKR1C3 inhibition by 3-4-trifluoromethyl-phenylamino-benzoic acid (10 μM) for 24 hours (gray bars) on adipose androgen generation, assessed as conversion of A4 to testosterone. (f) Impact of pharmacologic AKR1C3 inhibition on A4-mediated *de novo* lipogenesis. All data are presented as the mean ± standard error of the mean of 3 to 5 experiments. Significance levels: *P < 0.05; **P < 0.01; ***P < 0.001 (analysis of variance with Tukey *post hoc* test).

AKR1C3 messenger RNA expression increased significantly in both SGBS and primary female subcutaneous adipocytes (n = 3) after exposure to insulin 20 nM (*P* = 0.03 and *P* = 0.04, respectively) [Fig. 5(a) and 5(c)], but not in primary female omental adipocytes (n = 3, *P* = 0.18). Similarly, AKR1C3 activity was significantly increased by insulin exposure at doses in SGBS cells and primary female subcutaneous adipocytes [Fig. 5(b) and 5(d)], but not in primary omental adipocytes.

Selective pharmacologic AKR1C3 inhibition with 3-4-trifluoromethyl-phenylamino-benzoic acid (10 μM for 24 hours) significantly reduced generation of active androgen T from the androgen precursor A4. This was observed in both control adipocytes and those treated with 10 nM insulin (100% for control *vs* 59% ± 3% for inhibitor, *P* < 0.0001; 131% ± 10% for 10 nM insulin *vs* 77% ± 16% for inhibitor, *P* = 0.02) [Fig. 5(e)]. Adipocytes treated with 100 nM A4 showed significantly increased *de novo* lipogenesis (179.4% ± 21.5%, *P* = 0.002); this effect was significantly attenuated by coincubation with the selective AKR1C3 inhibitor (*P* = 0.04) [Fig. 5(f)]. Hence, disruption of AKR1C3-mediated androgen generation ameliorates androgen-mediated adverse effects on adipocyte lipogenesis, thereby mechanistically linking AE and insulin resistance.

## Discussion

Using a human *in vivo* physiology approach, we have demonstrated an adipose tissue-driven mechanistic link between AE and lipid metabolism in PCOS. We used mass spectrometry to determine both systemic and adipose tissue androgen concentrations, revealing significantly increased androgen synthesis in PCOS adipose tissue, alongside increased expression and activity of the androgen-activating enzyme AKR1C3. This demonstrates the important capacity of adipose tissue to act as a source of AE in PCOS. Furthermore, androgens promoted *in vitro* lipid accumulation in human adipocytes, and suppressed *in vivo* lipolysis in women with PCOS. The net effect of this is promotion of adipocyte hypertrophy. Enlarged, lipid-overloaded adipocytes are insulin resistant (32), fueling the adverse metabolic phenotype observed in PCOS, with lipid overspill and lipotoxicity in other organs such as the liver. We could show that *in vitro* inhibition of AKR1C3 decreased AE and subsequently *de novo* lipogenesis, identifying AKR1C3 as a target for therapeutic intervention in PCOS.

Several 17*β*-hydroxysteroid dehydrogenase enzymes are capable of activating A4 to T, but of those, only AKR1C3 is expressed in human adipose tissue (22). Interestingly, expression of the androgen-inactivating enzyme HSD17B2, which is capable of inactivating T to A4 and has been detected in omental adipose tissue (33), was not detectable in subcutaneous adipose tissue in this study or in previous work by our group (22). Our studies reveal the androgen-activating enzyme AKR1C3 as the predominant driver of active androgen generation in adipose tissue of PCOS patients. AKR1C3 is a crucial enzyme in androgen biosynthesis in the adrenal, ovary, prostate, and adipose tissue (34), and *AKR1C3* single nucleotide polymorphisms have been reported as associated with an increased prevalence of PCOS and hyperandrogenism in women (35). BMI differed slightly between the control and PCOS groups in this study (median, 33.1 *vs* 28.6 kg/m^2^); however, this difference did not show statistical significance, possibly because of the size of the cohorts. With this minor caveat in mind, our finding of increased AKR1C3 expression and activity confirm and expand the significance of results from a previous study describing increased AKR1C3 messenger RNA expression in subcutaneous adipose tissue of PCOS women (23).

In our *in vitro* studies, we conclusively demonstrated that AKR1C3 expression is regulated by insulin, mechanistically linking AE and insulin resistance, the two major metabolic features in PCOS. We found that insulin increased AKR1C3 expression and T generation *in vitro*, and *in vivo* we found a significant correlation between adipose tissue T and circulating insulin. Some previously published evidence had provided preliminary evidence for the potential regulation of adipose androgen metabolism by insulin. The fasting-induced transcription factor Kruppel-like factor 15 (KLF15) is crucially required for adipocyte differentiation (36), represents a key regulator of gluconeogenesis (37), and has been recently shown to represent a molecular link between glucose metabolism and lipogenesis (38). The AKR1C3 promoter contains a KLF15 binding site, and *in vitro* overexpression of KLF15 results in increased AKR1C3 promoter activity and T formation by adrenal NCIH295R cells (39). Intriguingly, we found increased expression of both AKR1C3 and KLF15 in the subcutaneous adipose tissue biopsies in our PCOS patients.

In this study, we demonstrated the functional impact of chronic and acute androgen exposure on the regulation of adipose tissue lipid metabolism, demonstrating suppressed lipolysis *in vivo* and increased lipogenesis *in vitro*. Data from nontargeted serum metabolomics support this observation of enhanced lipogenesis in PCOS. Serum metabolomics also revealed significantly increased concentrations of GPLs and LGPLs in women with PCOS but not in controls; the acute androgen challenge yielded a further increase of these metabolites in PCOS patients, whereas concentrations in BMI-matched controls decreased. This demonstrates distinctly different metabolic responses to androgens in PCOS and controls and suggests that AE plays a distinct role in creating a lipotoxic metabolic environment. Both GPLs and LGPLs previously identified as markers of risk and progression in nonalcoholic fatty liver disease (40), which is more prevalent in women with PCOS.

**Figure 6.**
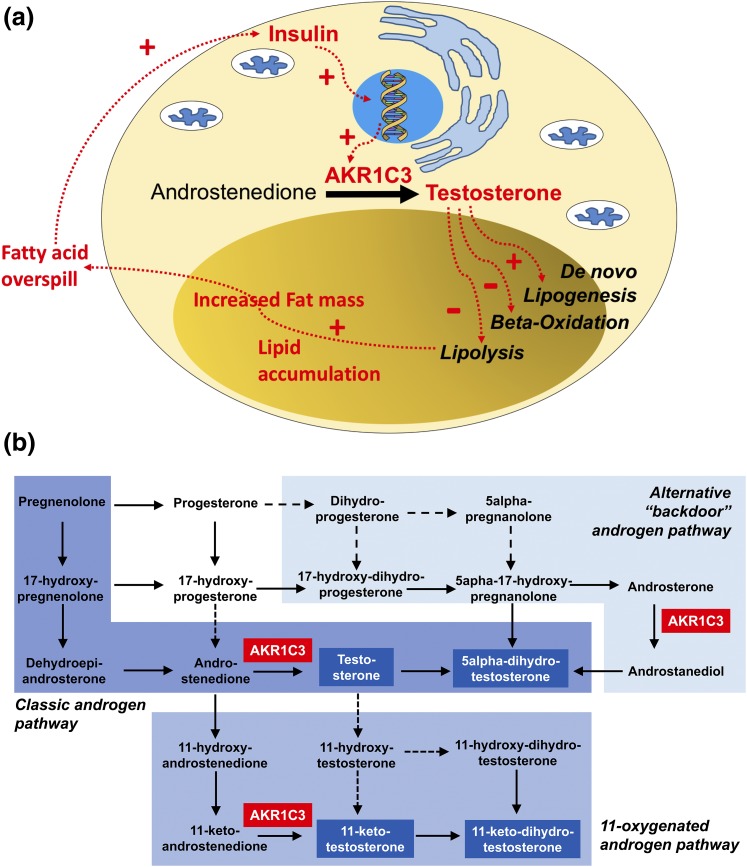
(a) Schematic representation of the proposed mechanistic link between AE, insulin resistance, and lipotoxicity in PCOS, and (b) graphical representation of the major human androgen biosynthesis pathways. AKR1C3 plays a central gatekeeping role in androgen activation in the classic androgen synthesis pathway and the alternative (backdoor) pathway to 5*α*-DHT and the 11-oxygenated androgen synthesis pathway. Active androgens capable of activating the androgen receptor highlighted in blue boxes and white font.

In conclusion, we have shown evidence that intra-adipose AE and dysfunctional adipose lipid metabolism are causally linked drivers of adverse metabolic risk in PCOS. We have described putative mechanisms by which androgens regulate adipose tissue function, lipid metabolism, and fat mass and have highlighted, at the level of the adipocyte, the enzyme AKR1C3 as a key regulator linking insulin resistance and AE in PCOS. We postulate that a vicious circle in adipose tissue drives metabolic risk in PCOS [Fig. 6(a)], consisting of increased androgen generation in adipose tissue by AKR1C3, subsequent lipid accumulation, and increased fat mass, resulting in systemic insulin resistance and lipotoxic organ damage, with androgen generation further exacerbated by hyperinsulinemia. We have shown that selective *in vitro* inhibition of AKR1C3 significantly reduced androgen-induced *de novo* lipogenesis. This suggests that local generation of T from A in subcutaneous adipose tissue by AKR1C3 is an important driver of androgen-mediated lipid accumulation.

The disruption of this vicious circle should now be studied in patients with PCOS, using selective pharmacologic inhibition of AKR1C3. Murine models are not suitable for the exploration of human androgen synthesis and metabolism as steroidogenic enzymes such as the 17*β*-hydroxysteroid dehydrogenase family are very differently organized in mice and humans. In humans, AKR1C3 is a central gatekeeper enzyme not only in classic androgen synthesis, but also in the alternative pathway to DHT synthesis (41) and the 11-oxygenated androgen pathway (42). Recent work (43) has shown that 11-oxygenated androgens represent most circulating androgens in PCOS, including 11-keto-T, which has three- to fourfold higher serum concentrations than T in PCOS patients, while activating the androgen receptor with similar potency. Therefore, selective AKR1C3 inhibition is likely to provide the most efficient control of PCOS-related AE [Fig. 6(b)]. In addition, based on our findings, inhibition of AKR1C3 is likely to exert beneficial effects on the adverse metabolic phenotype in PCOS, through amelioration of both circulating androgen burden and abnormal adipose tissue biology. In conclusion, AKR1C3 represents a highly promising therapeutic target in women with PCOS.

## References

[1] Randeva HS, Tan BK, Weickert MO, Lois K, Nestler JE, Sattar N, Lehnert H Cardiometabolic aspects of the polycystic ovary syndrome. Endocr Rev. 2012;33(5):812–841. 22829562 10.1210/er.2012-1003 PMC3461136

[2] Franks S Polycystic ovary syndrome. N Engl J Med. 1995;333(13):853–861. 7651477 10.1056/NEJM199509283331307

[3] Yildiz BO, Bozdag G, Yapici Z, Esinler I, Yarali H Prevalence, phenotype and cardiometabolic risk of polycystic ovary syndrome under different diagnostic criteria. Hum Reprod. 2012;27(10):3067–3073. 22777527 10.1093/humrep/des232

[4] Dunaif A Insulin resistance and the polycystic ovary syndrome: mechanism and implications for pathogenesis. Endocr Rev. 1997;18(6):774–800. 9408743 10.1210/edrv.18.6.0318

[5] Roe A, Hillman J, Butts S, Smith M, Rader D, Playford M, Mehta NN, Dokras A Decreased cholesterol efflux capacity and atherogenic lipid profile in young women with PCOS. J Clin Endocrinol Metab. 2014;99(5):E841–E847. 24512495 10.1210/jc.2013-3918 PMC4010695

[6] Legro RS, Kunselman AR, Dodson WC, Dunaif A Prevalence and predictors of risk for type 2 diabetes mellitus and impaired glucose tolerance in polycystic ovary syndrome: a prospective, controlled study in 254 affected women. J Clin Endocrinol Metab. 1999;84(1):165–169. 9920077 10.1210/jcem.84.1.5393

[7] Zhao L, Zhu Z, Lou H, Zhu G, Huang W, Zhang S, Liu F Polycystic ovary syndrome (PCOS) and the risk of coronary heart disease (CHD): a meta-analysis. Oncotarget. 2016;7(23):33715–33721. 27220885 10.18632/oncotarget.9553 PMC5085114

[8] Macut D, Tziomalos K, Božić-Antić I, Bjekić-Macut J, Katsikis I, Papadakis E, Andrić Z, Panidis D Non-alcoholic fatty liver disease is associated with insulin resistance and lipid accumulation product in women with polycystic ovary syndrome. Hum Reprod. 2016;31(6):1347–1353. 27076501 10.1093/humrep/dew076

[9] Jones H, Sprung VS, Pugh CJ, Daousi C, Irwin A, Aziz N, Adams VL, Thomas EL, Bell JD, Kemp GJ, Cuthbertson DJ Polycystic ovary syndrome with hyperandrogenism is characterized by an increased risk of hepatic steatosis compared to nonhyperandrogenic PCOS phenotypes and healthy controls, independent of obesity and insulin resistance. J Clin Endocrinol Metab. 2012;97(10):3709–3716. 22837189 10.1210/jc.2012-1382

[10] Rotterdam ESHRE/ASRM-Sponsored PCOS Consensus Workshop Group Revised 2003 consensus on diagnostic criteria and long-term health risks related to polycystic ovary syndrome. Fertil Steril. 2004;81(1):19–25. 10.1016/j.fertnstert.2003.10.004 14711538

[11] O’Reilly MW, Taylor AE, Crabtree NJ, Hughes BA, Capper F, Crowley RK, Stewart PM, Tomlinson JW, Arlt W Hyperandrogenemia predicts metabolic phenotype in polycystic ovary syndrome: the utility of serum androstenedione. J Clin Endocrinol Metab. 2014;99(3):1027–1036. 24423344 10.1210/jc.2013-3399 PMC3955250

[12] Stewart PM, Shackleton CH, Beastall GH, Edwards CR 5 alpha-reductase activity in polycystic ovary syndrome. Lancet. 1990;335(8687):431–433. 1968168 10.1016/0140-6736(90)90664-q

[13] Franks S, Gharani N, Gilling-Smith C Polycystic ovary syndrome: evidence for a primary disorder of ovarian steroidogenesis. J Steroid Biochem Mol Biol. 1999;69(1-6):269–272. 10419001 10.1016/s0960-0760(99)00044-8

[14] Moran C, Reyna R, Boots LS, Azziz R Adrenocortical hyperresponsiveness to corticotropin in polycystic ovary syndrome patients with adrenal androgen excess. Fertil Steril. 2004;81(1):126–131. 14711555 10.1016/j.fertnstert.2003.07.008

[15] Fassnacht M, Schlenz N, Schneider SB, Wudy SA, Allolio B, Arlt W Beyond adrenal and ovarian androgen generation: increased peripheral 5 alpha-reductase activity in women with polycystic ovary syndrome. J Clin Endocrinol Metab. 2003;88(6):2760–2766. 12788885 10.1210/jc.2002-021875

[16] Semple RK, Savage DB, Cochran EK, Gorden P, O’Rahilly S Genetic syndromes of severe insulin resistance. Endocr Rev. 2011;32(4):498–514. 21536711 10.1210/er.2010-0020

[17] Kim MS, Ryabets-Lienhard A, Dao-Tran A, Mittelman SD, Gilsanz V, Schrager SM, Geffner ME Increased abdominal adiposity in adolescents and young adults with classical congenital adrenal hyperplasia due to 21-hydroxylase deficiency. J Clin Endocrinol Metab. 2015;100(8):E1153–E1159. 26062016 10.1210/jc.2014-4033 PMC4524992

[18] Noordam C, Dhir V, McNelis JC, Schlereth F, Hanley NA, Krone N, Smeitink JA, Smeets R, Sweep FC, Claahsen-van der Grinten HL, Arlt W Inactivating PAPSS2 mutations in a patient with premature pubarche. N Engl J Med. 2009;360(22):2310–2318. 19474428 10.1056/NEJMoa0810489

[19] Oostdijk W, Idkowiak J, Mueller JW, House PJ, Taylor AE, O’Reilly MW, Hughes BA, de Vries MC, Kant SG, Santen GW, Verkerk AJ, Uitterlinden AG, Wit JM, Losekoot M, Arlt W PAPSS2 deficiency causes androgen excess via impaired DHEA sulfation--in vitro and in vivo studies in a family harboring two novel PAPSS2 mutations. J Clin Endocrinol Metab. 2015;100(4):E672–E680. 25594860 10.1210/jc.2014-3556 PMC4399300

[20] O’Reilly MW, House PJ, Tomlinson JW Understanding androgen action in adipose tissue. J Steroid Biochem Mol Biol. 2014;143:277–284. 24787657 10.1016/j.jsbmb.2014.04.008

[21] Blouin K, Blanchette S, Richard C, Dupont P, Luu-The V, Tchernof A Expression and activity of steroid aldoketoreductases 1C in omental adipose tissue are positive correlates of adiposity in women. Am J Physiol Endocrinol Metab. 2005;288(2):E398–E404. 15494612 10.1152/ajpendo.00312.2004

[22] Quinkler M, Sinha B, Tomlinson JW, Bujalska IJ, Stewart PM, Arlt W Androgen generation in adipose tissue in women with simple obesity--a site-specific role for 17beta-hydroxysteroid dehydrogenase type 5. J Endocrinol. 2004;183(2):331–342. 15531721 10.1677/joe.1.05762

[23] Wang L, Li S, Zhao A, Tao T, Mao X, Zhang P, Liu W The expression of sex steroid synthesis and inactivation enzymes in subcutaneous adipose tissue of PCOS patients. J Steroid Biochem Mol Biol. 2012;132(1-2):120–126. 22381227 10.1016/j.jsbmb.2012.02.003

[24] Hazlehurst JM, Oprescu AI, Nikolaou N, Di Guida R, Grinbergs AE, Davies NP, Flintham RB, Armstrong MJ, Taylor AE, Hughes BA, Yu J, Hodson L, Dunn WB, Tomlinson JW Dual-5α-reductase inhibition promotes hepatic lipid accumulation in man. J Clin Endocrinol Metab. 2016;101(1):103–113. 26574953 10.1210/jc.2015-2928 PMC4701851

[25] van Houten EL, Kramer P, McLuskey A, Karels B, Themmen AP, Visser JA Reproductive and metabolic phenotype of a mouse model of PCOS. Endocrinology. 2012;153(6):2861–2869. 22334715 10.1210/en.2011-1754

[26] Alexanderson C, Eriksson E, Stener-Victorin E, Lystig T, Gabrielsson B, Lönn M, Holmäng A Postnatal testosterone exposure results in insulin resistance, enlarged mesenteric adipocytes, and an atherogenic lipid profile in adult female rats: comparisons with estradiol and dihydrotestosterone. Endocrinology. 2007;148(11):5369–5376. 17656458 10.1210/en.2007-0305

[27] Varlamov O, White AE, Carroll JM, Bethea CL, Reddy A, Slayden O, O’Rourke RW, Roberts CT Jr Androgen effects on adipose tissue architecture and function in nonhuman primates. Endocrinology. 2012;153(7):3100–3110. 22547568 10.1210/en.2011-2111 PMC3380299

[28] Chadwick CA, Owen LJ, Keevil BG Development of a method for the measurement of dehydroepiandrosterone sulphate by liquid chromatography-tandem mass spectrometry. Ann Clin Biochem. 2005;42(Pt 6):468–474. 16259799 10.1258/000456305774538175

[29] Tomlinson JW, Moore JS, Clark PM, Holder G, Shakespeare L, Stewart PM Weight loss increases 11beta-hydroxysteroid dehydrogenase type 1 expression in human adipose tissue. J Clin Endocrinol Metab. 2004;89(6):2711–2716. 15181046 10.1210/jc.2003-031376 PMC7611657

[30] Arlt W, Justl HG, Callies F, Reincke M, Hübler D, Oettel M, Ernst M, Schulte HM, Allolio B Oral dehydroepiandrosterone for adrenal androgen replacement: pharmacokinetics and peripheral conversion to androgens and estrogens in young healthy females after dexamethasone suppression. J Clin Endocrinol Metab. 1998;83(6):1928–1934. 9626121 10.1210/jcem.83.6.4850

[31] Fischer-Posovszky P, Newell FS, Wabitsch M, Tornqvist HE Human SGBS cells - a unique tool for studies of human fat cell biology. Obes Facts. 2008;1(4):184–189. 20054179 10.1159/000145784 PMC6452113

[32] Kim JI, Huh JY, Sohn JH, Choe SS, Lee YS, Lim CY, Jo A, Park SB, Han W, Kim JB Lipid-overloaded enlarged adipocytes provoke insulin resistance independent of inflammation. Mol Cell Biol. 2015;35(10):1686–1699. 25733684 10.1128/MCB.01321-14 PMC4405637

[33] Fouad Mansour M, Pelletier M, Boulet MM, Mayrand D, Brochu G, Lebel S, Poirier D, Fradette J, Cianflone K, Luu-The V, Tchernof A Oxidative activity of 17β-hydroxysteroid dehydrogenase on testosterone in male abdominal adipose tissues and cellular localization of 17β-HSD type 2. Mol Cell Endocrinol. 2015;414:168–176. 26123590 10.1016/j.mce.2015.06.016

[34] Nakamura Y, Hornsby PJ, Casson P, Morimoto R, Satoh F, Xing Y, Kennedy MR, Sasano H, Rainey WE Type 5 17beta-hydroxysteroid dehydrogenase (AKR1C3) contributes to testosterone production in the adrenal reticularis. J Clin Endocrinol Metab. 2009;94(6):2192–2198. 19336506 10.1210/jc.2008-2374 PMC2690420

[35] Ju R, Wu W, Fei J, Qin Y, Tang Q, Wu D, Xia Y, Wu J, Wang X Association analysis between the polymorphisms of HSD17B5 and HSD17B6 and risk of polycystic ovary syndrome in Chinese population. Eur J Endocrinol. 2015;172(3):227–233. 25422294 10.1530/EJE-14-0615

[36] Mori T, Sakaue H, Iguchi H, Gomi H, Okada Y, Takashima Y, Nakamura K, Nakamura T, Yamauchi T, Kubota N, Kadowaki T, Matsuki Y, Ogawa W, Hiramatsu R, Kasuga M Role of Krüppel-like factor 15 (KLF15) in transcriptional regulation of adipogenesis. J Biol Chem. 2005;280(13):12867–12875. 15664998 10.1074/jbc.M410515200

[37] Gray S, Wang B, Orihuela Y, Hong EG, Fisch S, Haldar S, Cline GW, Kim JK, Peroni OD, Kahn BB, Jain MK Regulation of gluconeogenesis by Krüppel-like factor 15. Cell Metab. 2007;5(4):305–312. 17403374 10.1016/j.cmet.2007.03.002 PMC1892530

[38] Takeuchi Y, Yahagi N, Aita Y, Murayama Y, Sawada Y, Piao X, Toya N, Oya Y, Shikama A, Takarada A, Masuda Y, Nishi M, Kubota M, Izumida Y, Yamamoto T, Sekiya M, Matsuzaka T, Nakagawa Y, Urayama O, Kawakami Y, Iizuka Y, Gotoda T, Itaka K, Kataoka K, Nagai R, Kadowaki T, Yamada N, Lu Y, Jain MK, Shimano H KLF15 enables rapid switching between lipogenesis and gluconeogenesis during fasting. Cell Reports. 2016;16(9):2373–2386. 27545894 10.1016/j.celrep.2016.07.069 PMC5031553

[39] Du X, Rosenfield RL, Qin K KLF15 Is a transcriptional regulator of the human 17beta-hydroxysteroid dehydrogenase type 5 gene. A potential link between regulation of testosterone production and fat stores in women. J Clin Endocrinol Metab. 2009;94(7):2594–2601. 19366843 10.1210/jc.2009-0139 PMC2708951

[40] Anjani K, Lhomme M, Sokolovska N, Poitou C, Aron-Wisnewsky J, Bouillot JL, Lesnik P, Bedossa P, Kontush A, Clement K, Dugail I, Tordjman J Circulating phospholipid profiling identifies portal contribution to NASH signature in obesity. J Hepatol. 2015;62(4):905–912. 25450212 10.1016/j.jhep.2014.11.002

[41] Arlt W, Walker EA, Draper N, Ivison HE, Ride JP, Hammer F, Chalder SM, Borucka-Mankiewicz M, Hauffa BP, Malunowicz EM, Stewart PM, Shackleton CH Congenital adrenal hyperplasia caused by mutant P450 oxidoreductase and human androgen synthesis: analytical study. Lancet. 2004;363(9427):2128–2135. 15220035 10.1016/S0140-6736(04)16503-3

[42] Pretorius E, Arlt W, Storbeck KH A new dawn for androgens: novel lessons from 11-oxygenated C19 steroids. Mol Cell Endocrinol. 2017;441:76–85. 27519632 10.1016/j.mce.2016.08.014

[43] O’Reilly MW, Kempegowda P, Jenkinson C, Taylor AE, Quanson JL, Storbeck KH, Arlt W 11-Oxygenated c19 steroids are the predominant androgens in polycystic ovary syndrome. J Clin Endocrinol Metab. 2017;102(3):840–848. 27901631 10.1210/jc.2016-3285 PMC5460696

