# Supplementary material for: AKR1C3-Mediated Adipose Androgen Generation Drives Lipotoxicity in Women With Polycystic Ovary Syndrome
Source: J Clin Endocrinol Metab. 2017 Jun 22;102(9):3327–39. doi: 10.1210/jc.2017-00947 (PMC5587066; doi:10.1210/jc.2017-00947)
Supplement: Supplementary file 1 [file jc.2017-00947.st1.docx]

**SUPPLEMENTARY TABLES**

**Suppl. Table 1: Baseline clinical, metabolic and biochemical characteristics of PCOS patients and controls.** All data are expressed as median and interquartile range (25^th^-75^th^ percentile).

| **Variable** | **Controls (n=10)** | **PCOS (n=10)** | **P-value** |
| --- | --- | --- | --- |
| Age (years) | 31.0 (28.0-33.2) | 28.0 (22.3-35.3) | 0.42 |
| Body Mass Index (kg/m^2^) | 28.6 (24.8-33.8) | 33.1 (30.2-37.2) | 0.13 |
| ***Body composition (DXA)*** | | | |
| Truncal fat mass (g) | 394 (171-861) | 1150 (600-1562) | **0.02** |
| Truncal fat mass (%) | 1.3 (0.7-2.1) | 2.7 (1.9-3.1) | **0.02** |
| ***Insulin sensitivity*** | | | |
| Fasting plasma glucose (mmol/l) | 4.2 (4.1-4.6) | 4.5 (4.4-4.7) | 0.51 |
| Fasting serum insulin (pmol/l) | 19.5 (7.4-22.7) | 60.8 (49.8-129.7) | **0.02** |
| HOMA-IR | 0.8 (0.4-2.5) | 5.5 (3.1-10.4) | **0.003** |
| HbA1C (mmol/mol) | 34.5 (33.5-37.5) | 36.5 (34.5-37.8) | 0.21 |
| ***Fasting lipids*** | | | |
| Total cholesterol (mmol/L) | 4.2 (3.8-4.9) | 4.5 (4.2-5.3) | 0.20 |
| HDL (mmol/L) | 1.4 (1.3-1.7) | 1.2 (0.9-1.5) | **0.08** |
| Triglycerides (mmol/L) | 0.7 (0.5-0.8) | 1.2 (0.7-1.8) | **0.01** |
| ***Serum androgens*** | | | |
| DHEAS (μmol/L) | 4.1 (2.9-7.5) | 6.5 (3.5-9.3) | 0.43 |
| DHEA (nmol/L) | 6.2 (4.0-16.3) | 25.5 (12.6-35.2) | **0.004** |
| Androstenedione (nmol/L) | 4.0 (2.1-9.4) | 8.9 (7.0-11.8) | **0.04** |
| SHBG (nmol/L) | 45.8 (33.1-62.3) | 30.7 (23.5-41.4) | **0.03** |
| Testosterone (nmol/L) | 0.1 (0.0-0.2) | 0.9 (0.1-1.5) | **0.03** |
| FAI (Tx100/SHBG) | 2.1 (1.4-4.6) | 5.1 (3.4-7.7) | **0.008** |
| ***Urinary steroid excretion*** | | | |
| Urinary androsterone (An) (μg/24h) | 1641 (1103-2444) | 2114 (1504-3914) | 0.17 |
| Urinary etiocholanolone (Et) (μg/24h) | 1316 (866-1883) | 1433 (1038-2497) | 0.50 |
| Urinary 11β-hydroxyandrosterone (μg/24h) | 224 (116-408) | 327 (200-554) | 0.14 |
| Urinary An/Et ratio  (5α-reductase activity) | 1.2 (0.8-1.5) | 1.7 (1.2-1.8) | **0.02** |
| Urinary 5α-THF/THF ratio  (5α-reductase activity) | 0.9 (0.8-1.1) | 1.2 (0.8-1.5) | 0.27 |

Abbreviations: DXA, dual x-ray absorptiometry; DHEA, dehydroepiandrosterone; DHEAS, dehydroepiandrosterone sulfate; FAI, free androgen index; HDL, high-density lipoprotein; HOMA-IR, homeostasis model assessment of insulin resistance; SHBG, sex hormone-binding globulin; THF, tetrahydrocortisol.

**Suppl. Table 2: Correlation analysis (Spearman’s Rho, all patients) between adipose tissue androgen concentrations and baseline demographic and metabolic data.** Abbreviations: BMI, body mass index; HOMA-IR, homeostatic model assessment of insulin resistance; DHEA, dehydroepiandrosterone; A4, androstenedione; T, testosterone; DHT, 5α-dihydrotestosterone. *p<0.05; **p<0.01; ***p<0.001.

|  | **Age** | **BMI** | **HOMA-IR** | **Fasting insulin** | **Adipose DHEA** | **Adipose A4** | **Adipose T** | **Adipose DHT** |
| --- | --- | --- | --- | --- | --- | --- | --- | --- |
| **Age** |  | -0.058 | -0.285 | -0.250 | -0.301 | -0.147 | -0.088 | -0.059 |
| **BMI** | -0.058 |  | 0.318 | 0.361 | 0.282 | 0.290 | 0.282 | 0.258 |
| **HOMA-IR** | -0.285 | 0.318 |  | 0.951*** | 0.507* | 0.541* | 0.188 | 0.443* |
| **Fasting insulin** | -0.250 | 0.361 | 0.951*** |  | 0.450 | 0.351 | 0.492* | 0.272 |
| **Adipose DHEA** | -0.301 | 0.282 | 0.507* | 0.450 |  | 0.914*** | 0.713*** | 0.828*** |
| **Adipose A4** | -0.147 | 0.290 | 0.541* | 0.351 | 0.914*** |  | 0.529* | 0.954*** |
| **Adipose T** | -0.088 | 0.280 | 0.188 | 0.492* | 0.713*** | 0.529* |  | 0.444* |
| **Adipose DHT** | -0.059 | 0.258 | 0.443* | 0.272 | 0.828*** | 0.954*** | 0.444* |  |

**Suppl. Table 3: Significant changes in the circulating metabolome after an acute androgen challenge (DHEA 100mg orally) in PCOS patients and BMI-matched controls.** Fold change ranges (median and interquartile range) of serum metabolites observed to be statistically significant (p<0.01) for 15 metabolites classes when comparing control subjects before and 150 minutes after DHEA, and PCOS subjects before and 150 minutes after DHEA administration (fold changes calculated as before/after DHEA.

| **Metabolite classes** | **Control** | **PCOS** |
| --- | --- | --- |
| Arginine metabolism | 1.18 (1.17-1.24) | - |
| Aromatic amino acid metabolism | 1.23 (1.22-1.28) | 1.19 (1.16-1.26) |
| Ceramides and sphingolipids | 1.24 (1.18-1.25) | 1.42 (1.32-1.47) |
| Diacylglycerides | 1.16 (1.14-1.17) | 1.12 (1.11-1.14) |
| Fatty acid glycosides | 1.22 (1.14-1.24) | - |
| Fatty acids | 1.17 (1.14-1.20) | 1.16 (1.13-1.20) |
| Glycerophospholipid metabolism | 1.26 (1.21-1.28) | 0.91 (0.89-1.14) |
| Lysoglycerophospholipids | 1.24 (1.19-1.27) | 0.88 (0.84-0.90) |
| Monoacylglycerides | 1.17 (1.16-1.21) | - |
| Oxidised fatty acids, prostaglandins, thromboxanes and leukotrienes | 1.28 (1.18-1.40) | 1.14 (1.12-1.19) |
| Peptides | 1.32 (1.26-1.52) | 1.23 (1.18-1.34) |
| Sterol and steroid metabolism | 1.15 (1.11-1.21) | 1.12 (1.10-1.17) |

**SUPPLEMENTARY METHODS**

***Clinical protocol***

Participants attended the National Institute of Health Research/Wellcome Trust Clinical Research Facility (NIHR/WTCRF) at 8am, after an overnight fast, for a day of integrated assessment (**Fig. 1A)**. Each patient provided a pre-collected 24-hour urine sample for urinary steroid metabolite analysis by gas chromatography/mass spectrometry (GC/MS). Dual-energy x-ray absorptiometry (DXA) was employed for assessment of body composition and fat distribution using Hologic Discovery/W DXA (software version Apex 3.0). Fasting blood samples were drawn for plasma glucose, insulin, free fatty acids (FFAs), total cholesterol, high-density lipoprotein (HDL), triglycerides, sex hormone-binding globulin and liver biochemistry.

At 9am, an adipose microdialysis catheter (CMA microdialysis) was inserted, under local anesthetic and aseptic technique, 10cm lateral to the umbilicus; after a flush sequence, baseline samples were collected for adipose androgens. Additional samples were collected every 30 minutes (0.3μL/min), from 10am until 2pm, for measurement of glycerol, pyruvate, lactate and glucose. A subcutaneous abdominal adipose fat biopsy was obtained on each patient as described in the main Methods section. Baseline serum samples were taken at 9.45am for measurement of T, A, dehydroepiandrosterone (DHEA) and dehydroepiandrosterone sulfate (DHEAS). At 10am, 100mg of the oral androgen precursor DHEA (25mg capsules, Olympian) was administered to each participant; serum samples were then drawn every 30 minutes for 4 hours for T, A, DHEA, DHEAS, insulin, FFAs and glucose.

***Serum metabolic profiling***

Two serum samples were each collected from 19 subjects (8 controls and 8 PCOS, each subject for serum metabolic phenotyping); (1) basal phase/no treatment and (2) 150 minutes after DHEA administration. Samples were thawed on ice for approximately 60 minutes and deproteinized by addition of 600μL of methanol (pre-chilled to -20°C for 24h; HPLC grade, Sigma-Aldrich Chromasolv) to 200μL of serum followed by vortexing (15 seconds), centrifugation (15-minutes, 13500 xg, 3°C) and drying of 600μL of the supernatant (Thermo Scientific Savant SpeedVac Concentrator SPD111V)). A single pooled QC sample was prepared by combining 120μL aliquots of each biological sample followed by vortex mixing for one minute. 200μL aliquots were prepared in an identical process as described above.

All samples were analyzed applying Ultra Performance Liquid Chromatography (Ultimate3000RS; Thermo Scientific, Hemel Hempstead, UK) interfaced to an electrospray mass spectrometry (Q Exactive, Thermo Scientific, Hemel Hempstead, UK). Samples were reconstituted in 100μL 80/20 methanol/water and analyzed with QC samples analyzed ten times at the start of the batch (for system equilibration and MS/MS data acquisition), followed by injection after every 5th sample and finally two QC samples were analyzed at the end of the analytical batch. Paired samples for each subject were analyzed consecutively with each subject randomized across a single analytical batch. The mass spectrometer was tuned and calibrated applying standard procedures and solutions as defined by Thermo Scientific. UHPLC separations were performed applying a Hypersil Gold C18 reversed phase column (100 x 2.1 mm, 1.9 mm) at a flow rate of 400 mL.min^-1^, temperature of 40°C and with two solvents: solvent A (HPLC grade water +0.1% formic acid) and solvent B (HPLC grade methanol +0.1% formic acid). A gradient elution was performed as follows: hold 100% A 0–1.5 min, 100% A–100% B 1.5–6 min curve 3, hold 100% B 6–12 min, 100% B–100% A 12–13 min curve 3, hold 100% A 13–15 min. Injection volume was 5µL. UHPLC eluent was introduced directly in to the electrospray mass spectrometer with source conditions as follows: spray voltage -4.0 kV (ESI-) and +4.5 kV (ESI+), sheath gas 30 arbitrary units, aux gas 15 arbitrary units, capillary voltage 35 V, tube lens voltage -100 V (ESI-) and +90 V (ESI+), capillary temperature 280°C, ESI heater temperature 300°C. Data were acquired in ion mode switching in the m/z range 100–1000 at a mass resolution of 35 000 (FWHM defined at m/z 200), with a scan speed of 0.4 s and an AGC setting of 1 x 10^6^.

Data were processed applying XCMS as described previously (1). All metabolites reporting a relative standard deviation (RSD) > 20% and which were detected in <70% of QC samples from injection nine onwards were removed. The resulting data matrix was analyzed applying univariate (Mann-Whitney U test or Wilcoxon signed rank test) after normalization to total ion current per sample. Metabolites were annotated applying PUTMEDID_LCMS (2) and where feasible by comparison of HCD MS/MS mass spectra acquired for QC samples to the mass spectral library mzCloud (<https://www.mzcloud.org/>). All metabolites were annotated according to level 2 as defined by the Metabolomics Standards Initiative (3). Statistically significant metabolites were grouped in to classes related to chemical structure or metabolic function similarity.

***Urinary steroid excretion by gas chromatography-mass spectrometry (GC-MS)***

Urinary steroid metabolite excretion was carried out by GC-MS. Total androgen excretion was calculated by the sum of urinary androsterone (An) and etiocholanolone (Et). Net systemic 5α-reductase activity was measured by the ratios of An to Et and 5α-tetrahydrocortisol to tetrahydrocortisol (5α-THF/THF). Total glucocorticoid metabolite excretion was calculated by the sum of 5α-THF + THF + tetrahydrocortisone (THE) + cortolones + cortols + cortisol + cortisone.

**Simpson-Golabi-Behmel syndrome (SGBS) pre-adipocyte cell line culture**

The human preadipocyte cell line SGBS was obtained from Professor Martin Wabitsch, University of Ulm, Germany. Cells were differentiated into adipocytes according to previously published protocols, and previous work has validated SGBS cells as a good model of human adipocyte biology. Proliferating preadipocytes were cultured in Dulbecco’s MEM/Nutrient Mixture F12 (DMEM/F12, Sigma, Poole, UK) supplemented with 10% fetal calf serum. At confluence, cells were differentiated for 14 days in chemically-defined medium (DMEM/F12 with 33μM biotin, 17μM pantothenate, trasnferrin 0.01mg/ml, insulin 20nM, cortisol 100nM, T3 0.2nM, dexamethasone 25nM, rosiglitazone 2μM and methyl-3-isobutylxanthine (IBMX) 250μM (all reagents Sigma Aldrich Ltd). The AKR1C3 inhibitor, 3-4-trifluoromethyl-phenylamino-benzoic acid (Merck Millipore, UK), was added to wells for 15 min at a concentration of 10μM before addition of A. AKR1C3 activity was determined by generation of T after incubation of cells with A200nM for 24 hours. The PI3-kinase inhibitor wortmannin (500nM) was added to wells 15 min before the addition of insulin in selected experiments.

***RNA extraction, reverse transcription and real-time PCR***

Total RNA was extracted using the Tri-Reagent system. RNA integrity, reverse transcription and real-time PCR were performed as described previously (48). Probes and primers were supplied as TaqMan Gene Expression assays (Life Technologies, Paisley, UK). All reactions were normalized against two housekeeping genes, 18S and GAPDH rRNA. mRNA expression levels were determined using an ABI sequence detection system (Perkin-Elmer Applied Biosystems, Warrington, UK). Data were expressed as the cycle number at which logarithmic PCR plots crossed a calculated threshold line (ct values), with subsequent calculation of Δct value [Δct= (ct of target gene) – (ct of housekeeping gene)]. Fold change was calculated by the formula [fold increase = 2-difference in Δct].

***Functional studies of lipid metabolism***

*De novo lipogenesis*

Lipogenesis was measured by the uptake of 1-[^14^C]-acetate into the lipid component of adipocytes. After differentiation for 14 days, SGBS cells were washed and cultured in serum-free media for 4 hours. Cells were then treated with androgens (T20-40nM and DHT 10-20nM) with and without insulin (5nM) in serum-free media for 18 hours. Hot (1-[^14^C], 0.12μCi/L) and cold (10μM) acetate were added to the treatment wells for a further 6 hours. Cells were then washed three times and scraped with 250μL cold phosphate-buffered saline (PBS); cell lysate was transferred to glass TLC tubes. The lipid fraction was recovered in Foch solvent and the solvent was then evaporated to dryness. Radioactivity retained in the cellular lipid was determined by scintillation counting and expressed as disintegrations per minute (DPM)/per well. Results were expressed as % change from controls.

*β-oxidation*

Rates of β-oxidation were measured by the conversion of [^3^H]-palmitate to [^3^H]-H_2_0 (49). At day 14 of differentiation, SGBS cells were cultured in low-glucose/0.5% BSA serum-free media for 6h. Cells were then incubated with 300μL of low-glucose/0.5% serum-free media with hot (1mCi/ml [^3^H]-palmitate) and cold palmitate to a final concentration of 100μM palmitate, and treated with (5nM) or without insulin and androgens, as described above, at 37°C. After 24 hours, incubation medium was recovered and precipitated with 600μL 10% acetic acid. The aqueous component of the mixture was recovered and extracted with 2.5mL of 2:1 methanol:chloroform solution and 1ml of 2mM KCl:HC; per well. Radioactivity was determined by scintillation counting and expressed as DPM/well. Results were expressed as % change from controls per well.

*Free fatty acid uptake*

Free fatty acid uptake was estimated by intracellular [^3^H]-palmitate accumulation. Differentiated SGBS cells were washed and cultured in low-glucose/0.5% BSA serum-free media for 6 hours. This was followed by addition of hot (1mCi/ml [^3^H]-palmitate) and cold palmitate to a final concentration of 100μM palmitate; wells were then treated with (5nM) or without insulin and androgens, as described above, at 37°C for 24h. After incubation, cell lysate was recovered and radioactivity determined by scintillation counting. Radioactivity was expressed as DPM/well, and results represented as % change from controls per well.

**SUPPLEMENTARY REFERENCES**

(1) Dunn WB, Broadhurst D, Brown M, Baker PN, Redman CWG, Kenny LC, Kell DB. Metabolic profiling of serum using Ultra Performance Liquid Chromatography and the LTQ-Orbitrap mass spectrometry system. J. Chromatogr. B. Analyt. Technol. Biomed. Life Sci. 2008;871(2):288–98.

(2) Brown M, Wedge DC, Goodacre R, Kell DB, Baker PN, Kenny LC, Mamas MA, Neyses L, Dunn WB. Automated workflows for accurate mass-based putative metabolite identification in LC/MS-derived metabolomic datasets. Bioinformatics 2011;27(8):1108–12.

(3) Sumner LW, Amberg A, Barrett D, Beale MH, Beger R, Daykin CA, et al. Proposed minimum reportingstandards for chemical analysis. Metabolomics, 2007, 3(3), 211–221.
